# Function of the SIRT3 mitochondrial deacetylase in cellular physiology, cancer, and neurodegenerative disease

**DOI:** 10.1111/acel.12538

**Published:** 2016-09-29

**Authors:** Aneesa Ansari, Md. Shahedur Rahman, Subbroto K. Saha, Forhad K. Saikot, Akash Deep, Ki‐Hyun Kim

**Affiliations:** ^1^ Department of Genetic Engineering and BiotechnologyJessore University of Science and TechnologyJessore7408Bangladesh; ^2^ Department of Stem Cell and Regenerative BiologyKonkuk University120 Neungdong‐RoSeoul05029Korea; ^3^ Central Scientific Instruments Organisation (CSIR‐CSIO)Sector 30 CChandigarh160030India; ^4^ Department of Civil & Environmental EngineeringHanyang University222 Wangsimni‐RoSeoul04763Korea

**Keywords:** aging, cancer, Silent Information Regulator 2, SIRT3, sirtuin

## Abstract

In mammals, seven members of the sirtuin protein family known as class III histone deacetylase have been identified for their characteristic features. These distinguished characteristics include the tissues where they are distributed or located, enzymatic activities, molecular functions, and involvement in diseases. Among the sirtuin members, SIRT3 has received much attention for its role in cancer genetics, aging, neurodegenerative disease, and stress resistance. SIRT3 controls energy demand during stress conditions such as fasting and exercise as well as metabolism through the deacetylation and acetylation of mitochondrial enzymes. SIRT3 is well known for its ability to eliminate reactive oxygen species and to prevent the development of cancerous cells or apoptosis. This review article provides a comprehensive review on numerous (noteworthy) molecular functions of SIRT3 and its effect on cancer cells and various diseases including Huntington's disease, amyotrophic lateral sclerosis, and Alzheimer's disease.

## Introduction

SIRT3 is a NAD+‐dependent protein deacetylase that is a member of the silent information regulator 2 (SIR2) family (Giralt and Villarroya, 2012). SIRT3 can exert controls on a wide range of important biological activities including regulation of nuclear gene expression, metabolic control (Shi *et al*., 2005), neuroprotection (Kong *et al*., 2010), cardiovascular disease, cancer (Alhazzazi *et al*., 2011b), and aging (Bellizzi *et al*., 2005). SIRT3 is the only sirtuin protein reported to affect human lifespan (Kong *et al*., 2010; Brown *et al*., 2013; Kincaid & Bossy‐Wetzel, 2013). As it is localized exclusively in mitochondria, SIRT3 can regulate characteristic mitochondrial processes like protein deacetylation (Lombard *et al*., 2007). Some other organs and active metabolic tissues, including brown adipose tissue (BAT), heart, and kidney, were also reported to accommodate significant concentrations of SIRT3 (Palacios *et al*., 2009).

Among the mitochondrial sirtuins, only SIRT3 possesses robust NAD+‐dependent deacetylase activity (Lombard *et al*., 2007). SIRT3 can undergo proteolytic cleavage upon translocation to the mitochondrion. Full‐length (FL) SIRT3 is proposed to be inactive until it is translocated to and proteolytically processed within the mitochondrion to an active 28 kDa protein (Iwahara *et al*., 2012). Cleavage of SIRT3 at the mitochondrial localization sequence is known to activate deacetylase activity (Smith *et al*., 2008).

SIRT3 influences energy metabolism processes (e.g., tricarboxylic acid cycle, respiratory chain, fatty acid β‐oxidation, and ketogenesis) by targeting the responsible enzymes (Giralt and Villarroya, 2012). It also controls the flow of mitochondrial oxidative pathways and, ultimately, the rate of reactive oxygen species (ROS) production. SIRT3‐mediated deacetylation activates enzymes responsible for reducing ROS in protective action against oxidative stress‐dependent phenomena or diseases such as cardiac hypertrophy, aging, cancer, cardiac dysfunction, and neural degeneration. SIRT3 also plays a role in multiple additional metabolic processes from acetate metabolism to BAT thermogenesis, often by controlling mitochondrial pathways through the deacetylation of target enzymes (Shi *et al*., 2005).

The role of SIRT3 in physiology is crucial and an interesting subject for research. Protein acetylation regulates global mitochondrial function (Kim *et al*., 2006). Research implementing SIRT3‐knockout (KO) mice revealed many significant aspects of SIRT3 in physiology (Ahn *et al*., 2008). SIRT3 interacts with FOXO3a to activate antioxidant genes like manganese superoxide dismutase (MnSOD) and catalase, whose gene products reduce ROS while positively affecting disorders like cardiac hypertrophy and interstitial fibrosis (Sundaresan *et al*., 2009). SIRT3‐knockout mice are prone to age‐related disorders like cancer, cardiac hypertrophy, and metabolic syndrome (Choudhury *et al*., 2011; Hirschey *et al*., 2011). Frequent opening of mitochondrial permeability transition pore (mPTP) can lead to mitochondrial dysfunction. Note that mPTP is regulated by cyclophilin D (CypD) which is deacetylated by SIRT3. The absence of SIRT3 in cardiac muscle becomes a stimulus to increase the opening of mPTP. Thus, SIRT3 KO mice was found to suffer from hampering of mitochondrial function in heart with the sign of aging (Hafner *et al*., 2010). SIRT3‐knockout rodents also develop pulmonary arterial hypertension (Paulin *et al*., 2014). SIRT3 regulates stress response in hematopoietic stem cells (HSC) and improves regenerative capacity in aged HSCs, while it controls tissue homeostasis as well (Brown *et al*., 2013). SIRT3 can also manage NAD+ levels to regulate mitochondrial function, offering protection against liver injury associated with fatty liver (Kendrick *et al*., 2011) and/or acute kidney injury (Morigi *et al*., 2015). Because SIRT3 KO mice cannot maintain SOD2 homoeostasis and ROS level, they suffer from mild endothelial dysfunction when fed with high cholesterol diet (Winnik *et al*., 2016).

This study aims to provide a comprehensive review of the most relevant biological and pathophysiological functions of SIRT3 such as cancer control, neuroprotection, DNA repair, enhanced longevity, energy homoeostasis, oxidative stress tolerance, and many other mechanisms. To this end, most important studies conducted in this field were compiled and reviewed with respect to essential functions of SIRT3.

## Molecular functions of SIRT3

**Figure 1:**
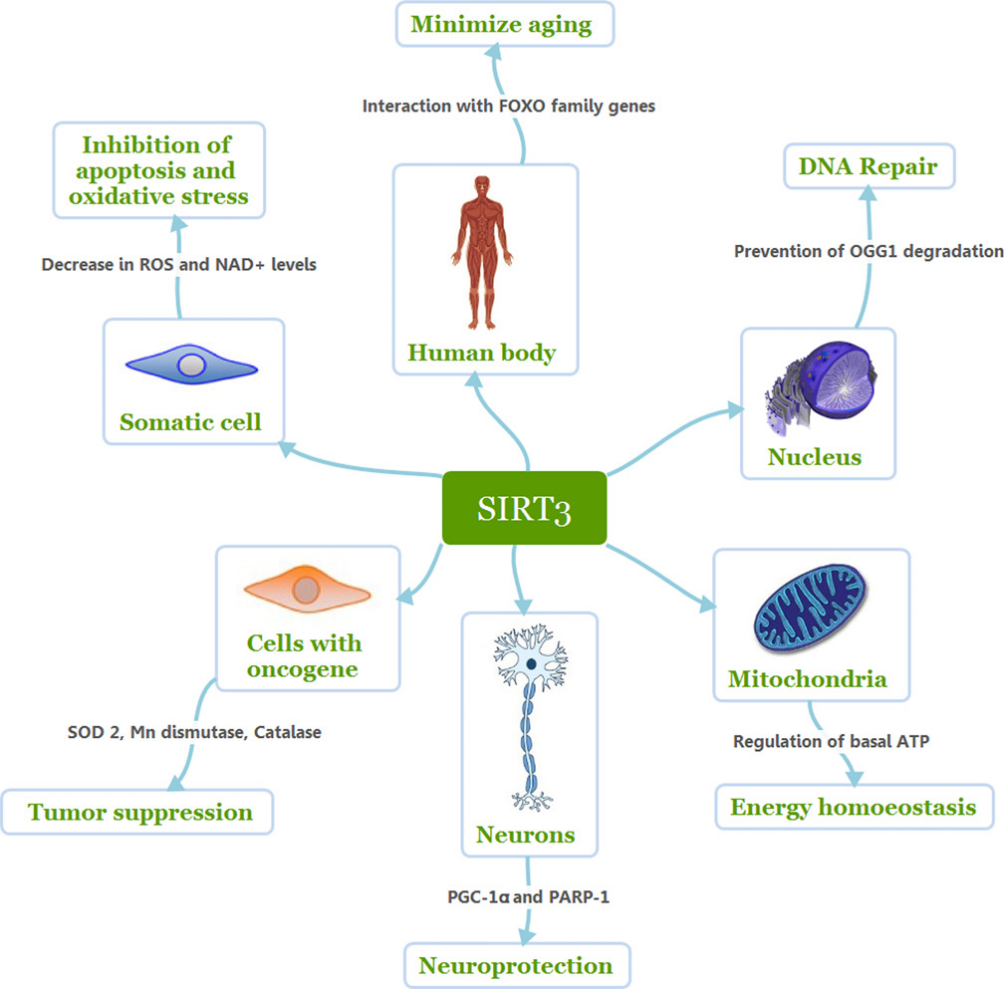
Molecular functions of SIRT3.

SIRT3 plays an important role in numerous molecular functions by controlling many crucial biological activities. As illustrated in Fig. 1, SIRT3 helps maintain the physiology of the body through various routes. (Refer to Table 1 for details.)

**Table 1:** Molecular functions of SIRT3

Molecular Function	Associated Enzyme/Protein	Activity of SIRT3	Location	Reference
Energy homeostasis	Absence of deacetylase	Reduction of ATP	Heart, kidney, liver	(Ahn *et al*., 2008)
	Protein acetylation	Increase in ATP	Mitochondria	
Suppression of ROS	Increase of superoxide dismutase 2	Stimulates SIRT3 transcription, leads to SOD2 deacetylation and activation of oxidative stress	Mitochondria	(Chen *et al*., 2011)
	Binds to peroxisome proliferator‐activated receptor co‐activator‐1α	Stimulates SIRT3 to regulate adaptive thermogenesis, gluconeogenesis, mitochondrial biogenesis, and respiration	Muscle cells and hepatocytes	(Lin *et al*., 2005; Finck & Kelly, 2006)
Apoptosis	SIRT3 along with SIRT4	Inhibits apoptosis by decreasing NAD+ levels	Somatic cells	(Yang *et al*., 2007)
	Mitochondrial ribosomal protein L10	Regulates mitochondrial protein synthesis by deacetylation of ribosomal protein MRPL10	Mitochondria	(Yang *et al*., 2010)
Tumor suppression	Decrease of superoxide dismutase	Depletion of SIRT3 leads to tumor suppression	Mitochondria	(Chen *et al*., 2011)
	Decrease of superoxide dismutase	Loss of SIRT3 promotes transformation through an increase in chromosomal instability via increased production of ROS and altered intracellular metabolism	Mitochondria	(Kim *et al*., 2010)
	Human 8‐oxoguanine‐DNA glycosylase 1	SIRT3 prevents the degradation of protein and repairs mitochondrial DNA damage	Mitochondria	(Cheng *et al*., 2013)
Muscles	Binds to Ku70 in cardiac muscles	Physically binds and deacetylates Ku70 and promotes interaction of Ku70 with the pro‐apoptotic protein Bax	Cardiomyocytes	(Sundaresan *et al*., 2008)
	CREB phosphorylation in skeletal muscles	Exercise increases SIRT3 expression	Skeletal muscle cells	(Palacios *et al*., 2009)
	Phospho‐activation of AMPK in skeletal muscles	SIRT3 expression increases with fasting and caloric restriction and decreases with high‐fat diet. A caloric restriction regimen also leads to phospho‐activation of AMPK in muscle	Skeletal muscle cells	(Palacios *et al*., 2009)
Neurons	LKB1	SIRT3 deacetylates and activates LKB1, augmenting LKB1–AMPK pathway activity, thus protecting against an increase in NAD	Mitochondria	(Miao & St Clair, 2009)
	Activates MnSOD and catalase	Decreases cellular levels of ROS	Mitochondria	(Kawamura *et al*., 2010)
	Poly (ADP‐ribose) polymerase‐1 activation or protein transfection of NADase	NAD depletion leads to over‐expression of SIRT3, which induces ROS generation, thereby preventing neuronal death	Mitochondria	(Kim *et al*., 2011)
Caloric restriction	Increase in the deacetylase	Induces the expression of genes involved in mitochondrial biogenesis	Adipocytes	(Shi *et al*., 2005)
Diabetes	Increase mitochondrial enzyme acetyl‐CoA synthetase 2	Increases SIRT3 under ketogenic conditions	Intermembrane space of the mitochondrion	(Schwer *et al*., 2006)
Metabolism	Activation of acetyl‐CoA synthetase 2 and glutamate dehydrogenase	Deacetylates and activates the enzymes to enhance the Krebs cycle and oxidative phosphorylation	Mitochondria	(Hallows *et al*., 2006; Schlicker *et al*., 2008)
	Succinate dehydrogenase complex	NAD+‐dependent deacetylase, SIRT3 regulates the activity of enzymes	Mitochondria	(Cimen, Han *et al*. 2009)
Thermogenesis	Increase expression of protein PGC‐1α, uncoupling protein (UCP1), and a series of mitochondria‐related genes in the presence of both ADP and ribosyltransferase	Deacetylates SIRT3	Brown adipose tissue and mitochondrial inner membrane	(Shi *et al*., 2005)
Fatty acid oxidation	Increase in long‐chain acyl‐CoA dehydrogenase	Upregulation of SIRT3	Fasting in liver and brown adipose tissues	(Allison & Milner, 2007)

### Significance of SIRT3 in metabolism

SIRT3 is characterized as a mitochondrial sirtuin protein that is localized in the mitochondrial matrix. It regulates the activity of several metabolic enzymes and Complex I subunit NDUFA9 by deacetylation (Ahn *et al*., 2008). Protein synthesis by oxidative phosphorylation (OXPHOS) is encoded by the mitochondrial genome, which is carried out by mitochondrial ribosomal protein L10 (MRP L10). However, MRP L10 generally remains in the acetylated form in the mitochondrial ribosome and becomes deacetylated by SIRT3 (Yang *et al*., 2010). SIRT3 deacetylates and activates acetyl‐CoA synthetase 2 and glutamate dehydrogenase (GDH), leading to enhancement of the Krebs cycle (Hallows *et al*., 2006; Schlicker *et al*., 2008). SIRT3 also regulates OXPHOS by activating acetyl‐CoA synthetase 2 and GDH (Ahn *et al*., 2008).

Keratinocyte differentiation is known to influence the formation/maintenance of the protective skin barrier (Bause *et al*., 2013; Chen *et al*., 2014). However, dysregulation in the balance of ROS homeostasis is known to impact keratinocyte differentiation. Loss of SIRT3 expression in keratinocytes increases superoxide levels and promotes the expression of differentiation markers, while overexpression decreases superoxide levels and reduces the expression of differentiation markers. However, it remains unclear how mitochondrial oxidative stress signaling induces and controls keratinocyte differentiation (Bause *et al*., 2013). Previous studies have shown that ROS signaling induces keratinocyte differentiation through the protein kinase C/activator protein‐1 (PKC/AP‐1) pathway (Rutberg *et al*., 1996; Bose *et al*., 2012). SIRT3 gene expression may in turn also be regulated through AP‐1 transcription factors as a possible regulatory pathway (Bellizzi *et al*., 2009). Cumulatively, SIRT3 is one of the most remarkable regulators in metabolism. It should be noted that the molecular control of SIRT3 itself is quite abstruse. The maintenance of mitochondrial DNA (mtDNA) is regulated by several components such as TFB1M and TFB2M which are known as very crucial factors determining the mitochondrial transcription specificity in human (Falkenberg *et al*., 2002). They are also regulated by nuclear respiratory factors (NRF1 and NRF2) (Gleyzer *et al*., 2005). For instance, as the role of NRF2 in the expression of SIRT3 promoter is significant, such mechanism can make substantial contribution to the mitochondrial biogenesis (Satterstrom *et al*., 2015).

### SIRT3 in the protection of neurons

One of the important functions of SIRT3 is the protection of neurons attained by the interaction with NAD+ and poly (ADP‐ribose) polymerase‐1 (PARP‐1). PARP‐1 is a DNA repair nuclear enzyme that helps prevent chromatid exchange (Schreiber *et al*., 2006). However, some stressful conditions like excitotoxicity, ischemia, inflammation, and oxidative stress cause over‐activation of PARP‐1, which is the key pathway responsible for neuronal cell death in the aforementioned consequences (Virág & Szabó, 2002). Nevertheless, over‐activation of PARP‐1 selectively decreases cytosolic NAD+ (Alano *et al*., 2004, 2010); PARP‐1‐mediated NAD+ depletion then accompanies colossal ROS production in the neuronal cell, which increases the expression of mitochondrial SIRT3. An overexpression of SIRT3 suppresses ROS generation, thereby preventing neuronal death (Kim *et al*., 2011). SIRT3 can also directly mediate adaptive neuronal responses to bioenergetic, oxidative, and excitatory stress. According to some experiments made with SIRT3 KO mice, cortical neurons lacking SIRT3 exhibited sensitivity to above‐mentioned physiological challenges. On the contrary, SIRT3 gene delivery can restore resistance to stress. The absence of SIRT3 was thus accompanied by hyperacetylation of SOD2 and CypD (Cheng *et al*., 2016).

**Figure 2:**
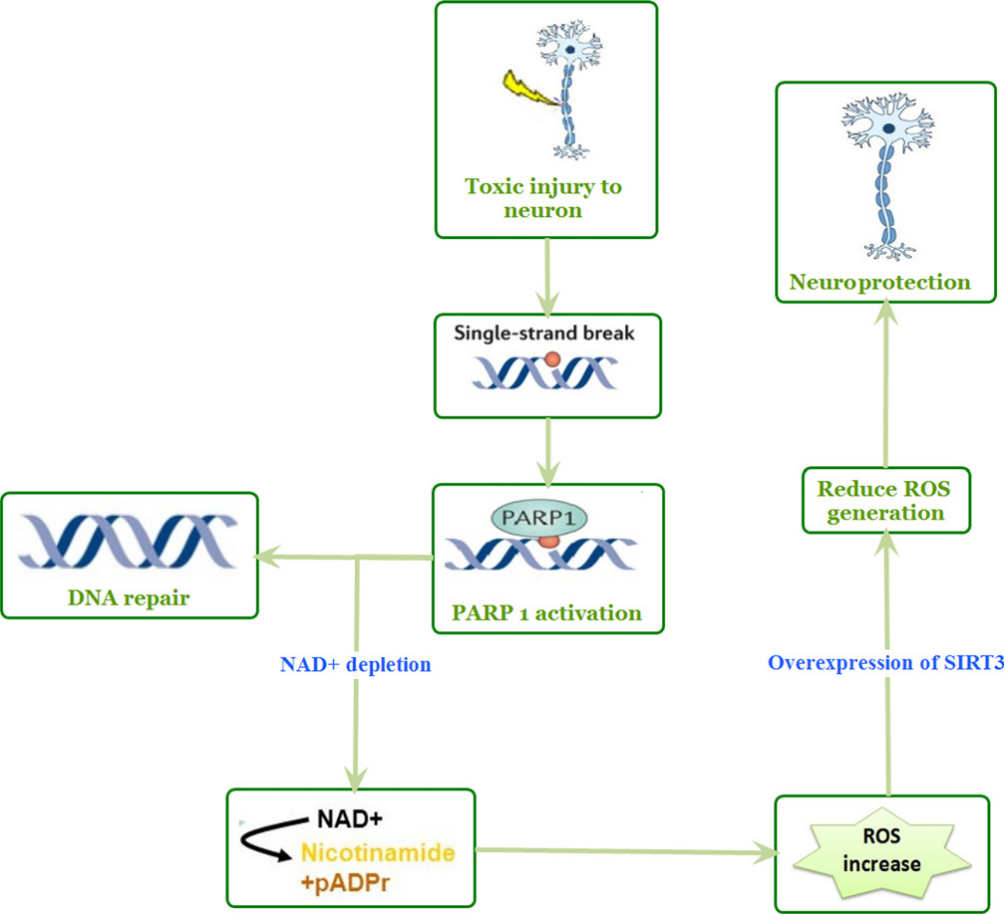
SIRT3 protect neurons during cellular stress.

Amyotrophic lateral sclerosis (ALS), an invariably fatal neurodegenerative disease, is characterized by the degeneration of both upper and lower motor neurons (Boillée *et al*., 2006; Zhao *et al*., 2013). Mutations in the related gene encoding Cu/Zn superoxide dismutase (SOD1) can affect an inherited form of ALS. Note that more than 150 types of *SOD1* gene mutations have been described, while most of them are transmitted in an autosomal dominant pattern (Battistini *et al*., 2005). The exact mechanism underlying SIRT3‐mediated protection against mutant SOD1‐induced toxicity remains elusive; however, the co‐presence of SIRT3 and peroxisome proliferator‐activated receptor‐γ coactivator‐1α (PGC‐1α) protects against mitochondrial fragmentation and neuronal cell death (Kong *et al*., 2010; Kincaid & Bossy‐Wetzel, 2013). The mitochondrial SIRT3 promoter region carries an estrogen‐related receptor (ERR)‐binding element (ERRE) located 399‐ to 407‐bp downstream (Kong *et al*., 2010). As oxidative stress increases, PGC‐1α recruits ERRα, a well‐known mitochondrial regulator, to the ERRE. The overexpression of SIRT3 is thus executed based on this mechanism (Kong *et al*., 2010). SIRT3 lowers ROS levels by deacetylating SOD2, while increasing its enzymatic activity (Bruijn *et al*., 2004; Qiu *et al*., 2010; Someya *et al*., 2010). Furthermore, SIRT1 is known to upregulate SIRT3 transcription through PGC‐1α (Tao *et al*., 2010; Giralt *et al*., 2011) (Fig. 2). PGC‐1α also co‐activates NRF2, another novel regulator of SIRT3 (Satterstrom *et al*., 2015). As neuroprotection by SIRT3 in above disease is mediated through PGC1α, it may help find future remedies of such disorders.

### Function of SIRT3 in different muscle types

**Figure 3:**
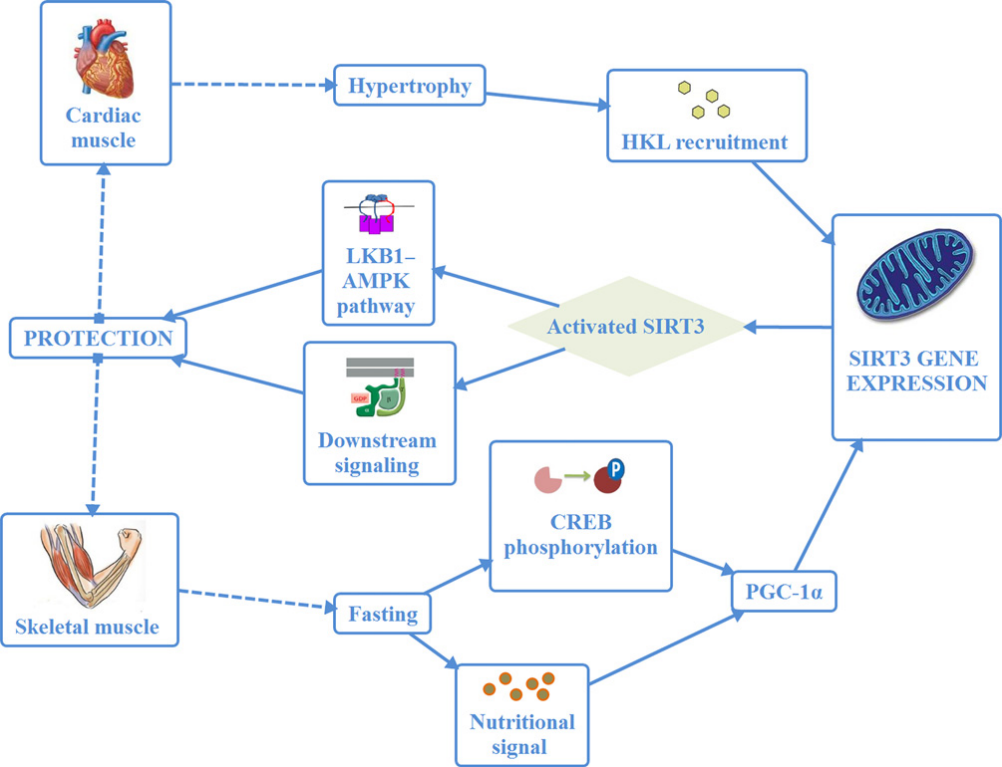
SIRT3‐mediated protection of cardiac and skeletal muscle during hypertrophy and fasting, respectively.

The expression of SIRT3 also plays a role in heart and skeletal muscles (Shi *et al*., 2005; Lombard *et al*., 2007). SIRT3 is a stress‐responsive deacetylase in cardiomyocytes and protects cells from genotoxic and oxidative stress‐mediated damage. As SIRT3 is a stress‐responsive deacetylase, its increased expression protects genotoxic and oxidative stress‐mediated cell death in murine cardiomyocytes. Under stressful conditions, SIRT3 levels increase in mitochondria as well as in cardiomyocyte nuclei. SIRT3 can then be physically bound to Ku70 for its deacetylation to promote interaction between Ku70 and the pro‐apoptotic protein Bax (Sundaresan *et al*., 2008). In addition, the antihypertrophic effects of exogenous NAD+ are also mediated through the activation of SIRT3 instead of SIRT1. As SIRT3 is able to deacetylate and activate liver kinase B1 (LKB1), it can augment LKB1–AMPK pathway activity. NAD+, while acting as an inhibitor of cardiac hypertrophic signaling, is regulated to protect against cardiac hypertrophy and heart failure (Miao & St Clair, 2009; Pillai *et al*., 2010). In mice, SIRT3 is activated by the natural biphenolic compound honokiol and protects cardiac muscles from hypertrophy (Fig. 3) (Sundaresan *et al*. 2009; Pillai *et al*., 2015).

In skeletal muscles, SIRT3 responds to both exercise and CREB phosphorylation with PGC‐1α upregulation of nutritional signals to coordinate downstream molecular responses (Palacios *et al*., 2009; Hokari *et al*., 2010) (Fig. 3). In skeletal muscle, SIRT3 protein level is also sensitive to diet as its expression increases by fasting and caloric restriction and decreases after a high‐fat diet. A caloric restriction regimen also leads to the phospho‐activation of AMPK in muscle (Palacios *et al*., 2009). When skeletal muscles were treated with near‐infrared light, an increase in oxidative stress was found with increases in the levels of upstream mitochondrial regulatory AMPK, p38, PGC‐1α, and SIRT1 proteins along with reduced levels of RIP140; however, mitochondrial regulation/content remained unaltered for SIRT3, Tfam, NRF‐1, cytochrome c, and ETC subunits (Nguyen *et al*., 2014). Consequently, the mechanism underlying such signaling in muscle cells remains unclear.

### SIRT3 in DNA repair

SIRT3 is capable of affecting mitochondrial OXPHOS by regulating the function of mitochondrial enzymes (Haigis *et al*., 2012). Human mitochondrial DNA (mtDNA) contains a number of genes that encode 13 polypeptides, two rRNAs, and 22 tRNA molecules; the 13 polypeptides are known to influence mitochondrial respiration and OXPHOS (Schon, 2000). Mutation of the mitochondrial genome may cause some inherited diseases; remarkable progress has been made from researches in mtDNA mutations with respect to aging and cancer (Taylor & Turnbull, 2005). Moreover, mtDNA is considerably more prone to oxidative damage than the nuclear genome (Richter *et al*., 1988). Oxygen‐free radicals generated by mitochondrial respiration or by exposure to ionizing radiation (or chemicals) are responsible for damaging DNA. Oxidative damage can thus change purine and pyrimidine bases in DNA to 8‐oxo‐7,8‐dihydroguanine (8‐oxoG) (Grollman & Moriya, 1993); however, human 8‐oxoguanine‐DNA glycosylase 1 (OGG1) is a newly identified target protein of SIRT3 that functions in DNA repair by expunging 8‐oxoG from damaged genomic DNA (Cheng *et al*., 2013). SIRT3 becomes physically associated with OGG1 to prevent degradation of the OGG1 protein and controls its incision activity when DNA glycosylase is acetylated. Furthermore, SIRT3 plays a critical role in repairing mitochondrial DNA (mtDNA) damage, protecting mitochondrial integrity, and preventing apoptotic cell death under oxidative stress by regulating the acetylation and turnover of OGG1 (Cheng *et al*., 2013).

### SIRT3 in aging

**Figure 4:**
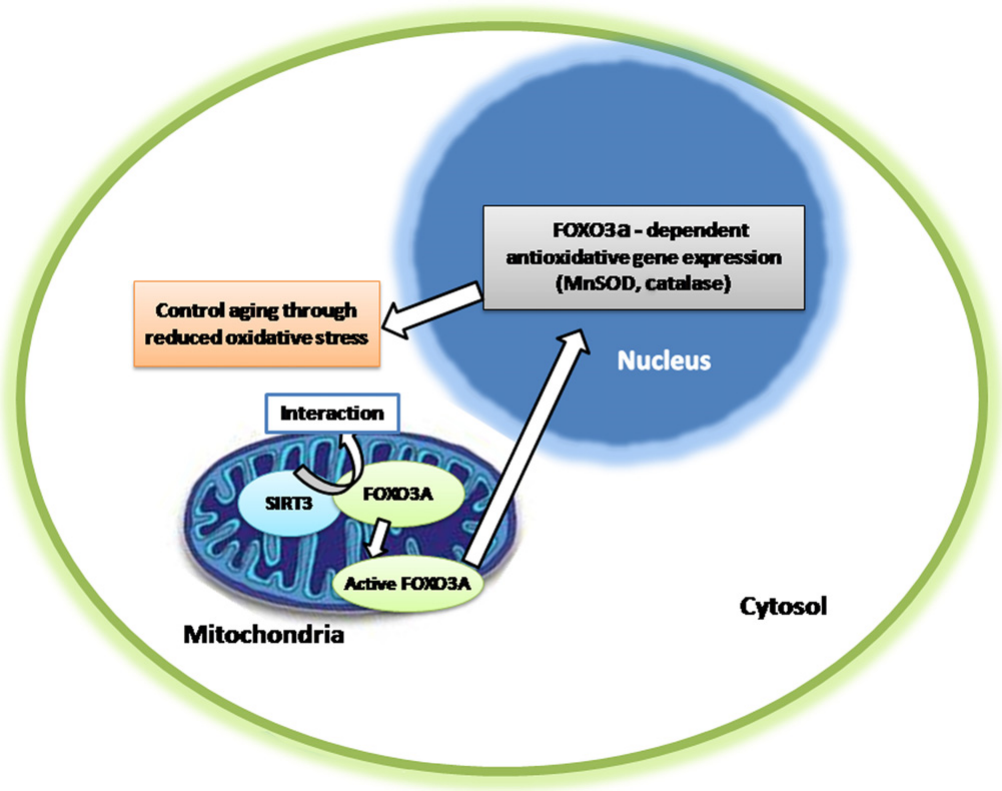
SIRT3 regulates aging by interacting with FOXO.

One of the most interesting attributes of SIRT3 is its potential to promote the extension of lifespan (Rose *et al*., 2003; Halaschek‐Wiener *et al*., 2009). A VNTR polymorphism prevails in the *SIRT3* gene as a potential regulator of SIRT3 expression. In light of the high abundance of SIRT3 in long‐lived individuals, a potential link was suggested between SIRT3 and longevity (Bellizzi *et al*., 2005). A better understanding on SIRT3‐dependence of expanded lifespan will indeed help broaden the spectrum of research in this field. The *FOXO1, FOXO3a, FOXO4,* and *FOXO6* genes encode the FOXO family of transcription factors and are human homologs of the *daf‐16* gene in *Caenorhabditis elegans*, the product of which contributes to the regulation of nematode lifespan (Shi *et al*., 2005). FOXO transcription factors appear to regulate a wide variety of intracellular processes such as cellular resistance to oxidative stress and general metabolism (Burgering & Kops, 2002; Accili & Arden, 2004). It is also acknowledged that nuclear sirtuins interact with the activity of FOXO family proteins under specific cellular conditions, as FOXO3a is known to physically interact with SIRT3 in mitochondria (Fig. 4) (Wang *et al*., 2007; Guarente, 2008). Human colon carcinoma (HCT116) cells were used to overexpress wild‐type and/or a catalytically inactive dominant species of negative *SIRT3* (Jacobs *et al*., 2008). Overexpression of the wild‐type *SIRT3* gene increases FOXO3a DNA‐binding activity and FOXO3a‐dependent gene expression. Biochemical analysis of HCT116 cells overexpressing a deacetylation mutant demonstrates an overall oxidized intracellular environment compared to overexpression of the wild‐type *SIRT3* gene, as monitored by increases in intracellular superoxide and oxidized glutathione levels (Wang *et al*., 2007). As such, both SIRT3 and FOXO3a follow a cascade response pathway for mitochondrial signaling (Jacobs *et al*., 2008). Tissue fibrosis caused by aging was reported to be mediated by an enzyme called glycogen synthase kinase 3β (GSK3β) (Frame & Cohen, 2001). The deacetylation of GSK3β by SIRT3 can thus potentially block aging process associated tissue fibrosis (Sundaresan *et al*., 2016).

SIRT3 can also interact with the mitochondrial acetyl‐coA synthetase known as AceCS2 (Allison & Milner, 2007). SIRT3/AceCS2 is required for G1 arrest that is induced by the loss of Bcl‐2 in a specific type of noncancer epithelial cells (ARPE19 cells). This event suggests a role of SIRT3/AceCS2 in apoptosis and growth regulation under certain environmental conditions (Allison & Milner, 2007). Although the molecular basis for lifespan extension is not yet clear, up‐to‐date knowledge on SIRT3 can stimulate scientists to seek exciting therapeutic solutions for aging.

### SIRT3 in energy homoeostasis

SIRT3 is an important regulator of basal ATP and controls overall energy homeostasis. Some tissues such as the heart, kidney, and liver normally express high levels of SIRT3, leading to a marked reduction in ATP in the absence of the deacetylase (Ahn *et al*., 2008). However, SIRT3 is capable of boosting the level of ATP in mitochondria due to the acetylation process. As mammalian mitochondrial ribosomal proteins are all nucleus‐encoded, some of them have been mapped to regions associated with disorders of mitochondrial energy metabolism (O'Brien *et al*., 2005). Alterations in the expression level and mutations in the genes encoding these ribosomal proteins can affect mitochondrial protein synthesis, cell growth, and apoptosis (Miller *et al*., 2004; Chintharlapalli *et al*., 2005; Yoo *et al*., 2005). Mitochondrial ribosomal protein L10 (MRPL10) is the major acetylated protein identified in the mitochondrial ribosome. Ribosome‐associated SIRT3 was thus found to be responsible for the NAD‐dependent deacetylation of MRPL10 (Yang *et al*., 2010). Mitochondrial biogenesis is also mandatory for oocyte development (John *et al*., 2010). *In vitro* maturation technique with metaphase II oocytes disclosed the developmental efficiency of SIRT3 by which mitochondrial energy homoeostasis and subsequent oocyte maturation are regulated. Hence, SiRNA‐induced SIRT3 knockdown precludes the biogenesis (Zhao *et al*., 2016).

### SIRT3 in oxidative stress

SIRT3 also plays a significant function in the regulation of ROS levels by influencing different enzymes like SOD2, manganese dismutase, and catalase (Miao & St Clair, 2009; Merksamer *et al*., 2013). As increases in ROS levels can stimulate *SIRT3* transcription, it may ultimately lead to deacetylation of SOD2 with the activation of oxidative stress (Chen *et al*., 2011); however, PGC‐1α plays a vital role in adaptive thermogenesis, gluconeogenesis, mitochondrial biogenesis, and respiration to induce ROS‐detoxifying enzymes (Lin *et al*., 2005; Finck & Kelly, 2006). Nonetheless, the molecular mechanism underlying this phenomenon is not yet fully understood. PGC‐1α and nuclear ERRα are known to strongly stimulate gene expression of mouse *SIRT3* in hepatocytes and muscle cells (Lin *et al*., 2005; Giralt and Villarroya, 2012).

### SIRT3 in stress‐related gene expression

Human SIRT3 exists in a full‐length (FL) form that is processed to a distinct short form to localize specifically to the mitochondria; hence, only the short form of human SIRT3 is active as an NAD+‐dependent deacetylase (Schwer *et al*., 2002). It was reported that FL SIRT3 may also be present in the nucleus and is capable of activating histone deacetylase (HDAC) against acetylated histone H3 Lys 9 (H3K9ac) and H4K16ac *in vivo* (Scher *et al*., 2007; Nakamura *et al*., 2008; Sundaresan *et al*., 2008). Such HDAC activity is believed to be associated with transcriptional repression when artificially recruited to a transgenic reporter (Tao *et al*., 2010). As nuclear FL SIRT3 is subject to cellular stress conditions (e.g., oxidative stress and UV irradiation), it can lead to rapid degradation without affecting the mitochondrial SIRT3 short form. Therefore, the rapid removal of SIRT3 from chromatin could induce genes required for the stress response. Binding of FL SIRT3 to chromatin could also suppress neighboring genes and mediate the deacetylation of H4K16 by activating ubiquitination and proteasome degradation (Iwahara *et al*., 2012). However, there have been some contradictions about the localization of SIRT3. Many reports nonetheless claimed that SIRT3 should be exclusively located at mitochondria (Michishita *et al*., 2005; Lombard *et al*., 2007; Cooper & Spelbrink, 2008).

### SIRT3 in thermogenesis

SIRT3 is also expressed in brown adipose tissue (BAT), where it is localized to the inner membranes of mitochondria (Shi *et al*., 2005). Caloric restriction activates SIRT3 expression in both BAT and brown adipose tissue (WAT). Unlike BAT, *SIRT3* gene expression does not occur in WAT upon cold exposure. In HIB1B brown adipocytes, an imposed expression of SIRT3 augments the expression of PGC‐1α, uncoupling protein 1 (UCP1), and a series of mitochondria‐related genes in the presence of both ADP‐ribosyltransferase and the deacetylase activity of SIRT3 (Shi *et al*., 2005). A SIRT3 deacetylase mutant has a synergistic effect with PGC‐1α to activate UCP1 expression in the mitochondrial inner membrane to mediate the process of adaptive thermogenesis. In addition, SIRT3 stimulates CREB phosphorylation to directly activate the PGC‐1α promoter. Sustained expression of SIRT3 decreases ROS production and membrane potential, while causing increased cellular respiration. *SIRT3* and other genes controlling mitochondrial function are downregulated in the BAT of several genetically obese mice (Shi *et al*., 2005). In the absence of SIRT3, high levels of fatty acid oxidation intermediate products and triglycerides are formed, while long‐chain acyl‐CoA dehydrogenase (LCAD) is hyperacetylated at Lys 42. LCAD can also be deacetylated under fasting conditions by SIRT3 as hyperacetylation of LCAD reduces its enzymatic activity (Allison & Milner, 2007; Hirschey *et al*., 2010).

### SIRT3 in tumor suppression

To date, the most remarkable feature of SIRT3 is its ability to suppress tumors and/or cancer. As a key regulator of different cancers, SIRT3 detoxifies ROS to act as a tumor suppressor (Schumacker, 2010; Tanno *et al*., 2012; Tseng *et al*., 2013). Under such conditions, mitochondrial MnSOD is an important antioxidant enzyme that is primarily regulated by transcriptional activation (Miao & St Clair, 2009). Decrease in enzymatic activity is caused by acetylation at Lys 68; however, mitochondrial deacetylase SIRT3 is able to bind and deacetylate for its activation. Increase in ROS levels also stimulates *SIRT3* transcription, leading to SOD deacetylation and activation. Thus, SOD‐mediated ROS reduction is synergistically increased by SIRT3 co‐expression, although it can be negated by SIRT3 depletion. As a result, a mechanism involving post‐translational regulation of SOD activity was revealed by elaborating the effect to oxidative stress on acetylation and SIRT3‐dependent deacetylation (Chen *et al*., 2011). However, deletion of *SIRT3* removes the requirement for the loss of a tumor suppressor for transformation of primary cells with an oncogene (Kim *et al*., 2010). SIRT3 KO mouse embryonic fibroblast (SIRT3^−/−^ MEF) demonstrates genomic instability as well as aberrant mitochondrial physiology. Single oncogenic expression (Myc or Ras) alters intracellular metabolism which is recovered by SOD (Kim *et al*., 2010). Contribution of SIRT3 in cancer genetics is discussed extensively in the following segment.

## The role of SIRT3 in cancer

**Figure 5:**
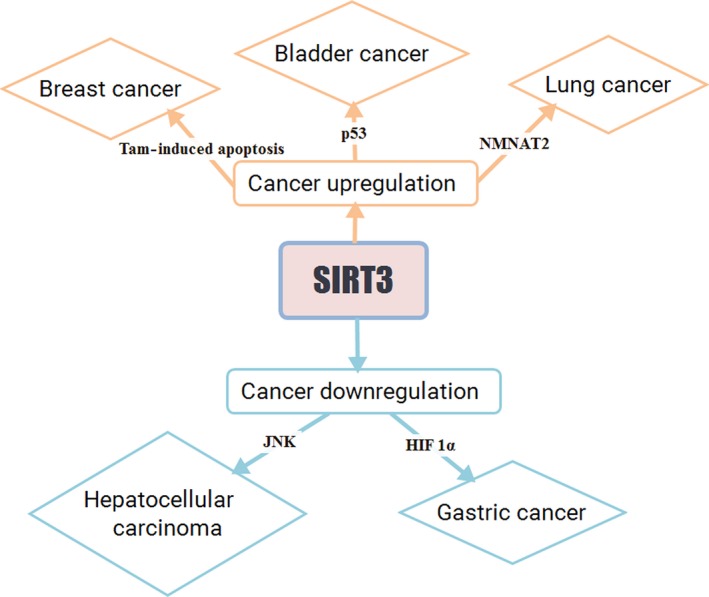
SIRT3 has both oncogenic and tumor suppressive activities.

The role of SIRT3 in human cancer has been studied intensively to validate its effect on the disease state. SIRT3 has the ability to reprogram cellular metabolism. In tumor cells, glycolysis occurs at a high rate even in the presence of O_2_, which is known as the Warburg effect (Haigis *et al*., 2012). Warburg effect helps tumor cells by supplementing with biomass‐generating substrates (Finley *et al*., 2011a). SIRT3 regulates the Warburg effect by decreasing the high glycolysis rate in tumor cells. Restricting tumorous cells from gaining substrates and/or biomass is achieved by the decreased level of glycolysis (Finley *et al*. 2011). Some of the important activities of SIRT3 in different cancers are discussed here and are overviewed in Table 2 and Fig. 5.

**Table 2:** Function of SIRT3 in different cancers

Type of cancer	Type of cells used	Activity of SIRT3	References
Oral cancer	Oral squamous cell carcinoma cell line	Cell growth and proliferation are inhibited by SIRT3 downregulation	(Alhazzazi *et al*., 2011a,b)
Breast cancer	MCF‐7 cell line produced through continuous selective culture in the presence of Tamoxifen	SIRT3 decreases apoptosis and decreases cellular sensitivity to Tamoxifen	(Zhang *et al*., 2013b)
Hepatocellular carcinoma	Hepatoma cells	Overexpression of SIRT3 leads to JNK activation and resulting apoptosis	(Zhang & Zhou, 2012)
Esophageal cancer	Patients with esophageal cancer	Increase in SIRT3 decreases ROS levels, prolonging survival rate	(Zhao *et al*., 2013)
Lung cancer	Nonsmall cell lung carcinoma	Low SIRT3 decreases cancer cell growth	(Li *et al*., 2013)
Gastric cancer	MGC‐803 gastric cancer cells	Presence of SIRT3 decreases cancer	(Huang *et al*., 2014)
Bladder cancer	p53 bladder carcinoma cells	SIRT3 prevents cancer cells from spreading	(Li *et al*., 2010)

### Oral cancer

According to a tissue microarray analysis, SIRT3 was overexpressed in oral squamous cell carcinoma (OSCC) relative to normal human oral keratinocytes cell lines (Alhazzazi *et al*., 2011a). Cell growth and proliferation are inhibited by a downregulation of SIRT3, while OSCC cell sensitivity increases with radiation and cisplatin treatments *in vitro*. SIRT3 downregulation reduces tumor burden *in vivo* (Alhazzazi *et al*., 2011a); however, significant decreases in SIRT3 mRNA and protein level were due to the effect of (‐)‐epigallocatechin‐3‐gallate, an antioxidative catechin commonly found in green tea (Tao & Lambert, 2014).

### Breast cancer

Tamoxifen (Tam) is commonly used as an adjuvant therapy during the management of breast cancer as it acts as an antagonist to the related selective estrogen receptor, that is, estrogen receptor‐positive (ER+). A study of the Tam‐resistant human breast cancer cell line (MTR‐3) revealed significant upregulation of SIRT3 at both the mRNA and protein levels (Zhang *et al*., 2013b). The above cell line was derived from the standard MCF‐7 cell line through a selective culture process in the presence of 1 mM Tam. SIRT3 level also rapidly increases in MCF‐7 cells after exposure to Tam. When the *SIRT3* gene is overexpressed in MCF‐7 cells, a decrease in cellular sensitivity to Tam is accompanied by a blockage in Tam‐induced apoptosis. Furthermore, cells are susceptible to Tam and apoptotic cell death when SIRT3 expression is knocked down in MTR‐3 cells. These MTR‐3 cells also showed increases in the mitochondrial content of ERb, ROS levels, and apoptosis (Zhang *et al*., 2013b). In addition to ER+ breast cancer, low SIRT3 expression is also associated with survival in the ER−, HER2+, and basal breast cancer subtypes. These results imply that SIRT3 can be used as a molecular biomarker to diagnose patients with high‐risk breast cancer (Desouki *et al*., 2014). Although some other sirtuin molecules like SIRT7 have impacts on breast cancer (Ashraf *et al*., 2006), little is known about the contribution of SIRT3. A form of human cancer known as luminal B breast cancer is suspected to have certain linkage with the inefficiency of SIRT3. SIRT3 KO murine models are found to develop tumors with similar characteristics of human luminal B breast cancer due probably to acetylation of MnSOD. In contrast, wild‐type mice did not show such symptoms, while MnSOD was deacetylated by SIRT3 (Zou *et al*., 2016).

### Hepatocellular carcinoma

SIRT3 protein expression in hepatocellular carcinoma (HCC) is associated with the degree of differentiation, pathological features, and complications with portal vein tumor thrombus. It is known that relative SIRT3 protein expression gradually decreases with the increases in differentiation between hepatoma cells and between the deterioration and progression of hepatocellular cells (Zhang *et al*., 2013a). The SIRT3 mechanism should thus be related to ROS levels in the mitochondria (Chen *et al*., 2011; Park *et al*., 2011; Schumacker, 2011); however, low SIRT3 expression was reported to act as a poor independent prognostic factor of survival in patients with postsurgical hepatocellular carcinoma (Zhang *et al*., 2012). The results of other research have provided evidence of increased recurrence with a decrease in SIRT3 level in HCC (Wang *et al*., 2014a). In hepatocellular carcinoma cells, overexpression of SIRT3 facilitated the activation of the JNK signaling pathway, resulting in apoptosis (Zhang & Zhou, 2012); however, low SIRT3 expression is associated with a markedly shorter period of clinical recurrence.

### Esophageal cancer

A number of studies have analyzed the clinical significance of SIRT3 expression in esophageal squamous cell carcinoma (ESCC). High SIRT3 expression levels in ESCC tissues were previously reported to occur more frequently than in adjacent nonmalignant esophageal mucosa tissues (Yan *et al*., 2014). The 5‐year postoperational survival rate of patients with esophageal cancer was examined in relation with the SIRT3 expression (Zhao *et al*., 2013). Accordingly, high expression of SIRT3 was related to a shorter survival time in patients with esophageal cancer, which might be due to the suppression of apoptosis signals that prolonged the survival rate of esophageal cancer. Moreover, tumors with high SIRT3 level might lead to genomic or signaling deregulation, taking advantage of SIRT3 overexpression to prolong survival by decreasing ROS levels (Zhao *et al*., 2013). Presumably, an overexpression of SIRT3 may help cancer cells divide and grow further. In addition, a reduction in cellular ROS is suggested to activate anti‐apoptotic proteins like Bcl‐2 and Bcl‐xL (Li *et al*., 2004).

### Lung cancer

**Figure 6:**
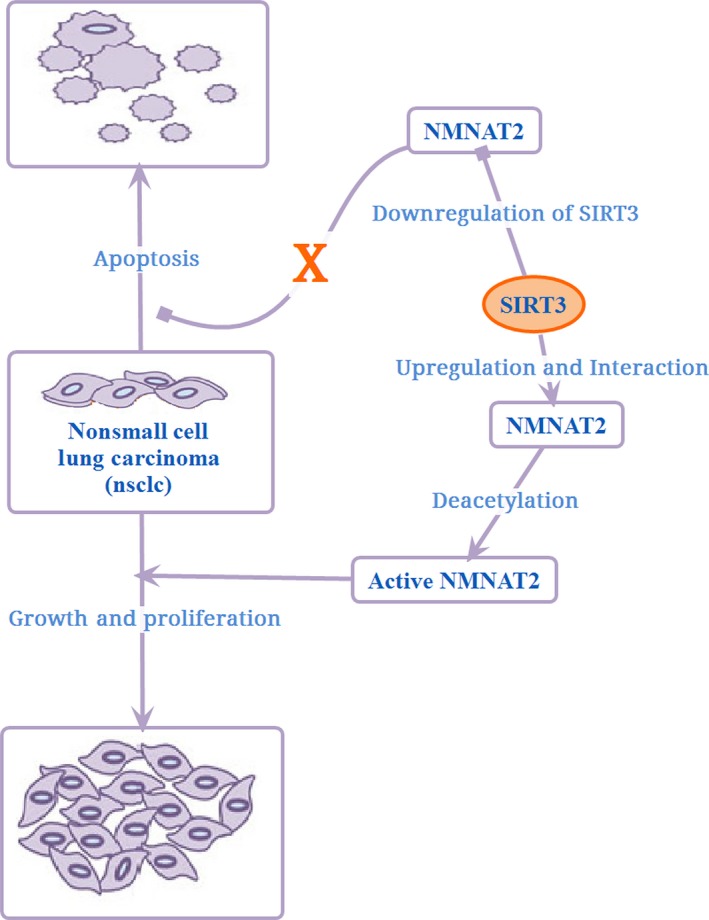
Function of SIRT3 in lung cancer.

Nonsmall cell lung carcinoma (NSCLC) is the most common lung cancer subtype encompassing approximately 85% of all such cases. Most of these patients have locally advanced or distant metastatic disease (stage III/IV) from the onset of symptoms. A 5‐year survival rate of NSCLC was reported to be less than 10% and 5% in male and female patients, respectively (Reungwetwattana *et al*., 2012). It was found that SIRT3 interacted with the full‐length nicotinamide mononucleotide adenylyl transferase 2 (*NMNAT2*) gene, causing its deacetylation to promote mitotic entry, growth, and proliferation of cultured cells *in vitro* and *in vivo*. Moreover, downregulation of SIRT3 apparently inhibited the acetylation of *NMNAT2* and NAD+ synthesis enzyme activity (Yan *et al*., 2010). However, a lack of SIRT3 expression caused the inhibition of mitotic entry, growth, and proliferation of a NSCLC cell line, leading to apoptosis. This is actually related to energy metabolism involved in the interaction between SIRT3 and *NMNAT2* (Li *et al*., 2013) (Fig. 6). *NMNAT2* is a promising therapeutic target in lung cancer treatment.

### Gastric cancer

SIRT3 also plays a role in gastric cancer (GC); however, its role in the pathogenesis of GC remains unclear. It has been reported that SIRT3 expression level is inversely correlated with such factors as tumor infiltration, tumor differentiation, and tumor stage. *In vitro* experiments showed that the absence of SIRT3 in MGC‐803 GC cells significantly increased the expression of HIF‐1a (Yang *et al*., 2014); therefore, aberrantly decreased expression of SIRT3 was seen in patients with GC. SIRT3 is likely to function as a mitochondrial tumor suppressor in GC to affect the progression of patients with GC by exerting direct control on HIF‐1a. However, it is unclear whether the aberrant expression of SIRT3 in patients with GC is a causal factor, a subsequent effect of tumor progression, or if other mutations play a role in GC (Wang *et al*., 2015). The Notch family of proteins substantially contributes to cell behavior including cell proliferation, differentiation, and apoptosis (Vallejo *et al*., 2011). Notch‐1 is also overexpressed in GC, suggesting its critical role in the disease (Du *et al*., 2014). SIRT3 suppresses the proliferation of GC cells through the downregulation of Notch‐1, which suggests a novel therapeutic target in GC therapy (Wang *et al*., 2015). Previously, it was observed that GC patients with SIRT3 expression have better prognosis than those without it. SIRT3 expression is an independent prognostic marker of survival to act as a tumor suppressor in GC (Huang *et al*., 2014).

### Bladder cancer

Extracellular/intracellular stress may lead to cell cycle arrest, apoptosis, and cellular senescence in bladder cancer (Li *et al*., 2012). Accordingly, tumor protein p53 functions to regulate the transcriptional program in the related events of above cellular stress. The mitochondrial p53 gene has an antiproliferative function, although it is normally expressed in the nucleus (Talos *et al*., 2005). In the absence of p53, an EJ bladder carcinoma cell line maintained growth arrest until p53 was introduced (Sugrue *et al*., 1997). Endogenous p53 can be independently partitioned with mitochondrial and nuclear proteins, as it is capable of promoting senescence. SIRT3 thus rescued p53‐EJ cells from induced p53‐mediated growth arrest and partially nullified the ability of p53 to enact growth arrest and senescence; however, after the introduction of the chaperone protein BAG‐2, SIRT3 targeting of p53‐mediated senescence was averted (Li *et al*., 2010).

## The role of SIRT3 in other diseases

Although SIRT3 has a potential role in cancer, it can also exert important control on other diseases. The most notable aspects of these diseases are described below.

### Alzheimer's disease

ROS marker analysis revealed the prevalence of oxidative stress in Alzheimer's disease (AD). This analysis was carried out by determining oxidized biomolecules generated by ROS (Wang *et al*., 2014b). Oxidative stress is engaged in a spiral interaction with mitochondrial dysfunction. Thus, it poses a major threat by producing ROS that are critical for the pathogenesis of AD as well as a number of other neurodegenerative diseases (Lin & Beal, 2006). Recent studies revealed that SIRT3 expression has been increasing in both human and mouse AD pathology. Apolipoprotein E_4_ (APOE_4_) is a major genetic factor in late‐onset AD, and SIRT3 is known to be downregulated in the frontal cortices of human brain APOE_4_ carriers compared to noncarriers. Analysis of SIRT3 level may aid in the diagnosis of AD (Yin *et al*., 2015). In addition, SIRT3 level may increase in response to increased ROS synthesis. In an experiment with primary neuronal cultures, the occurrence of such SIRT3 upregulation indicated a possibility for an increased oxidative stress‐oriented mitochondrial response in AD. An increase in LF SIRT3 could well extend the neuronal lifespan under mitochondrial oxidative stress (Weir *et al*., 2012).

### Huntington's disease

Huntington's disease (HD) as a progressive and autosomal dominant neurodegenerative disorder develops with psychiatric manifestations, cognitive decline, and movement abnormalities (Harper, 1996). HD is caused by abnormal polyglutamine expression in the Huntingtin (Htt) protein (Naia & Rego, 2015). To date, there is no cure for HD. Nonetheless, as energy‐demanding neurons are particularly susceptible to energy deficits and oxidative stress, mitochondrial dysfunction might be a mediating factor for the mutant Htt‐induced neurotoxicity (Fu *et al*., 2012). It was previously reported that cells expressing mutant Htt displayed reduced SIRT3 levels, and mutant Htt‐induced depletion of SIRT3 protected cells from mutant Htt. The natural product viniferin and other semisynthetic stilbenic compounds can also decrease ROS levels; as such, they will decrease deacetylase activity along with a reduction in cellular NAD+ levels and mitochondrial biogenesis in cells. Viniferin also activates AMP‐activated kinase while enhancing mitochondrial biogenesis. On the other hand, a knockdown significantly inhibited viniferin‐mediated AMP‐activated kinase activation with diminished neuroprotective effects (Fu *et al*., 2012). Thus, SIRT3 is suggested to mediate the neuroprotection of viniferin (Fu *et al*., 2012).

### Amyotrophic lateral sclerosis

The ALS disease shows an increase in fragmented mitochondria along with defects to bi‐directional axonal transport with increased cell death. In a model of amyotrophic lateral sclerosis (ALS), SIRT3 is able to prevent mitochondrial fragmentation of spinal cord motor neurons transfected with SOD1^G93A^. Interestingly, co‐expression with either SIRT3 or PGC‐1α is able to rescue SOD1^G93A^‐induced mitochondrial fragmentation to improve cell survival (Song *et al*., 2013). SIRT3 regulates ketone body production by deacetylation of mitochondrial 3‐hydroxy‐3‐methylglutaryl CoA synthase 2 (HMGCS2), indicating its potential for neuroprotection in the SOD1^G93A^ model (Pasinetti *et al*., 2013).

### Age‐related hearing loss

Age‐related hearing loss (AHL) is frequently explained in association with oxidative stress. In mice, oxidative stress causes damage to hair cells and spiral ganglia neurons of the cochlea, which results in hearing loss in about 12 months. Calorie restriction induces SIRT3, which prevents or delays age‐related hearing loss by protecting cochlear neurons from oxidative damage (Someya *et al*., 2010; Bell & Guarente, 2011). Mitochondria can maintain oxidative stress by the glutathione antioxidant system that is mediated by increased levels of NADPH, which is achieved by direct deacetylation of mitochondrial isocitrate dehydrogenase 2 (IDH2) by SIRT3 (Someya *et al*., 2010; Jing *et al*., 2011).

## Conclusion

SIRT3 is a NAD+‐dependent protein deacetylase. It is a noble protein with numerous roles that are essential in human physiology. Starting with energy homeostasis and metabolism, SIRT3 impacts nuclear and muscular function, reduces the effects of aging, and mediates several genetic diseases. Moreover, it has the ability to repair DNA, regulate thermogenesis, and fight against oxidative stress. SIRT3 has been noted extensively for its role in different types of cancer such as oral cancer, breast cancer, esophageal cancer, lung cancer, gastric cancer, and hepatocellular carcinoma and also in other diseases such as AD, HD, ALS, and AHL. Perceptual research in the field of SIRT3 is continually increasing our knowledge. Both *in vitro* and *in vivo* studies can be used to acquire information regarding the potential of different SIRT3 activators and the pharmacological applications of this protein. In summary, SIRT3 is a clinically novel target for various complications of human physiology.

## Conflict of Interest

The authors have no conflict of interests to declare.

## References

[acel12538-bib-0001] Accili D , Arden KC (2004) FoxOs at the crossroads of cellular metabolism, differentiation, and transformation. Cell 117, 421–426. 15137936 10.1016/s0092-8674(04)00452-0

[acel12538-bib-0002] Ahn B‐H , Kim H‐S , Song S , Lee IH , Liu J , Vassilopoulos A , Deng C‐X , Finkel T (2008) A role for the mitochondrial deacetylase Sirt3 in regulating energy homeostasis. Proc. Natl. Acad. Sci. USA 105, 14447–14452. 18794531 10.1073/pnas.0803790105 PMC2567183

[acel12538-bib-0003] Alano CC , Ying W , Swanson RA (2004) Poly (ADP‐ribose) polymerase‐1‐mediated cell death in astrocytes requires NAD+ depletion and mitochondrial permeability transition. J. Biol. Chem. 279, 18895–18902. 14960594 10.1074/jbc.M313329200

[acel12538-bib-0004] Alano CC , Garnier P , Ying W , Higashi Y , Kauppinen TM , Swanson RA (2010) NAD+ depletion is necessary and sufficient for poly (ADP‐Ribose) polymerase‐1‐mediated neuronal death. J. Neurosci. 30, 2967–2978. 20181594 10.1523/JNEUROSCI.5552-09.2010 PMC2864043

[acel12538-bib-0006] Alhazzazi TY , Kamarajan P , Joo N , Huang JY , Verdin E , D'Silva NJ , Kapila YL (2011a) Sirtuin‐3 (SIRT3), a novel potential therapeutic target for oral cancer. Cancer 117, 1670–1678. 21472714 10.1002/cncr.25676 PMC3117020

[acel12538-bib-0007] Alhazzazi TY , Kamarajan P , Verdin E , Kapila YL (2011b). SIRT3 and cancer: tumor promoter or suppressor? Biochim. Biophys. Acta. 1816, 80–88. 21586315 10.1016/j.bbcan.2011.04.004 PMC3129516

[acel12538-bib-0008] Allison SJ , Milner J (2007) SIRT3 is pro‐apoptotic and participates in distinct basal apoptotic pathways. Cell Cycle 6, 2669–2677. 17957139 10.4161/cc.6.21.4866

[acel12538-bib-0009] Ashraf N , Zino S , Macintyre A , Kingsmore D , Payne A , George W , Shiels P (2006) Altered sirtuin expression is associated with node‐positive breast cancer. Br. J. Cancer 95, 1056–1061. 17003781 10.1038/sj.bjc.6603384 PMC2360714

[acel12538-bib-0010] Battistini S , Giannini F , Greco G , Bibbò G , Ferrera L , Marini V , Causarano R , Casula M , Lando G , Patrosso MC (2005) SOD1 mutations in amyotrophic lateral sclerosis. J. Neurol. 252, 782–788. 15789135 10.1007/s00415-005-0742-y

[acel12538-bib-0011] Bause AS , Matsui MS , Haigis MC (2013) The protein deacetylase SIRT3 prevents oxidative stress‐induced keratinocyte differentiation. J. Biol. Chem. 288, 36484–36491. 24194516 10.1074/jbc.M113.472324 PMC3868761

[acel12538-bib-0012] Bell EL , Guarente L (2011) The SirT3 divining rod points to oxidative stress. Mol. Cell 42, 561–568. 21658599 10.1016/j.molcel.2011.05.008 PMC3526939

[acel12538-bib-0013] Bellizzi D , Rose G , Cavalcante P , Covello G , Dato S , De Rango F , Greco V , Maggiolini M , Feraco E , Mari V , Franceschi C , Passarino G , De Benedictis G (2005) A novel VNTR enhancer within the SIRT3 gene, a human homologue of SIR2, is associated with survival at oldest ages. Genomics 85, 258–263. 15676284 10.1016/j.ygeno.2004.11.003

[acel12538-bib-0014] Bellizzi D , Covello G , Di Cianni F , Tong Q , De Benedictis G (2009) Identification of GATA2 and AP‐1 Activator elements within the enhancer VNTR occurring in intron 5 of the human SIRT3 gene. Mol. Cells 28, 87–92. 19714312 10.1007/s10059-009-0110-3

[acel12538-bib-0015] Boillée S , Vande Velde C , Cleveland DW (2006) ALS: a disease of motor neurons and their nonneuronal neighbors. Neuron 52, 39–59. 17015226 10.1016/j.neuron.2006.09.018

[acel12538-bib-0016] Bose A , Teh M‐T , Hutchison IL , Wan H , Leigh IM , Waseem A (2012) Two mechanisms regulate keratin K15 expression in keratinocytes: role of PKC/AP‐1 and FOXM1 mediated signalling. PLoS One 7, e38599. 22761689 10.1371/journal.pone.0038599 PMC3384677

[acel12538-bib-0017] Brown K , Xie S , Qiu X , Mohrin M , Shin J , Liu Y , Zhang D , Scadden DT , Chen D (2013) SIRT3 reverses aging‐associated degeneration. Cell Rep. 3, 319–327. 23375372 10.1016/j.celrep.2013.01.005 PMC3582834

[acel12538-bib-0018] Bruijn LI , Miller TM , Cleveland DW (2004) Unraveling the mechanisms involved in motor neuron degeneration in ALS. Annu. Rev. Neurosci. 27, 723–749. 15217349 10.1146/annurev.neuro.27.070203.144244

[acel12538-bib-0019] Burgering BM , Kops GJ (2002) Cell cycle and death control: long live Forkheads. Trends Biochem. Sci. 27, 352–360. 12114024 10.1016/s0968-0004(02)02113-8

[acel12538-bib-0020] Chen Y , Zhang J , Lin Y , Lei Q , Guan KL , Zhao S , Xiong Y (2011) Tumour suppressor SIRT35 deacetylates and activates manganese superoxide dismutase to scavenge ROS. EMBO Rep. 12, 534–541. 21566644 10.1038/embor.2011.65 PMC3128277

[acel12538-bib-0021] Chen I , Chiang WF , Chen PF , Chiang HC (2014) STRESS‐responsive deacetylase SIRT3 is up‐regulated by areca nut extract‐induced oxidative stress in human oral keratinocytes. J. Cell. Biochem. 115, 328–339. 24339251 10.1002/jcb.24667

[acel12538-bib-0022] Cheng Y , Ren X , Gowda AS , Shan Y , Zhang L , Yuan Y , Patel R , Wu H , Huber‐Keener K , Yang J (2013) Interaction of Sirt3 with OGG1 contributes to repair of mitochondrial DNA and protects from apoptotic cell death under oxidative stress. Cell Death Dis. 4, e731. 23868064 10.1038/cddis.2013.254 PMC3730425

[acel12538-bib-0023] Cheng A , Yang Y , Zhou Y , Maharana C , Lu D , Peng W , Liu Y , Wan R , Marosi K , Misiak M (2016) Mitochondrial SIRT3 mediates adaptive responses of neurons to exercise and metabolic and excitatory challenges. Cell Metab. 23, 128–142. 26698917 10.1016/j.cmet.2015.10.013 PMC5141613

[acel12538-bib-0024] Chintharlapalli SR , Jasti M , Malladi S , Parsa KV , Ballestero RP , González‐García M (2005) BMRP is a Bcl‐2 binding protein that induces apoptosis. J. Cell. Biochem. 94, 611–626. 15547950 10.1002/jcb.20292

[acel12538-bib-0025] Choudhury M , Jonscher KR , Friedman JE (2011) Reduced mitochondrial function in obesity‐associated fatty liver: SIRT3 takes on the fat. Aging (Albany NY) 3, 175–178. 21386135 10.18632/aging.100289 PMC3082013

[acel12538-bib-0200] Cimen H , Han MJ , Yang Y , Tong Q , Koc H , Koc EC (2009) Regulation of succinate dehydrogenase activity by SIRT3 in mammalian mitochondria. Biochemistry 49, 304–311. 10.1021/bi901627u PMC2826167 20000467

[acel12538-bib-0026] Cooper HM , Spelbrink JN (2008) The human SIRT3 protein deacetylase is exclusively mitochondrial. Biochemical J. 411, 279–285. 10.1042/BJ20071624 18215119

[acel12538-bib-0027] Desouki MM , Doubinskaia I , Gius D , Abdulkadir SA (2014) Decreased mitochondrial SIRT3 expression is a potential molecular biomarker associated with poor outcome in breast cancer. Hum. Pathol. 45, 1071–1077. 24746213 10.1016/j.humpath.2014.01.004 PMC4030591

[acel12538-bib-0028] Du X , Cheng Z , Wang Y‐H , Guo Z‐H , Zhang S‐Q , Hu J‐K , Zhou Z‐G (2014) Role of Notch signaling pathway in gastric cancer: a meta‐analysis of the literature. World J. Gastroenterol. 20, 9191. 25083094 10.3748/wjg.v20.i27.9191 PMC4112896

[acel12538-bib-0029] Falkenberg M , Gaspari M , Rantanen A , Trifunovic A , Larsson N‐G , Gustafsson CM (2002) Mitochondrial transcription factors B1 and B2 activate transcription of human mtDNA. Nat. Genet. 31, 289–294. 12068295 10.1038/ng909

[acel12538-bib-0030] Finck BN , Kelly DP (2006) PGC‐1 coactivators: inducible regulators of energy metabolism in health and disease. J. Clin. Investig. 116, 615–622. 16511594 10.1172/JCI27794 PMC1386111

[acel12538-bib-0031] Finley LW , Carracedo A , Lee J , Souza A , Egia A , Zhang J , Teruya‐Feldstein J , Moreira PI , Cardoso SM , Clish CB , Pandolfi PP , Haigis MC (2011) SIRT3 opposes reprogramming of cancer cell metabolism through HIF1α destabilization. Cancer Cell 19, 416–428. 21397863 10.1016/j.ccr.2011.02.014 PMC3065720

[acel12538-bib-0032] Frame S , Cohen P (2001) GSK3 takes centre stage more than 20 years after its discovery. Biochemical J. 359, 1–16. 10.1042/0264-6021:3590001 PMC1222116 11563964

[acel12538-bib-0033] Fu J , Jin J , Cichewicz RH , Hageman SA , Ellis TK , Xiang L , Peng Q , Jiang M , Arbez N , Hotaling K (2012) trans‐(−)‐ϵ‐Viniferin increases mitochondrial sirtuin 3 (SIRT3), activates AMP‐activated protein kinase (AMPK), and protects cells in models of Huntington Disease. J. Biol. Chem. 287, 24460–24472. 22648412 10.1074/jbc.M112.382226 PMC3397871

[acel12538-bib-0005] Giralt A , Villarroya F (2012) SIRT3, a pivotal actor in mitochondrial functions: metabolism, cell death and aging. Biochemical J. 444, 1–10. 10.1042/BJ20120030 22533670

[acel12538-bib-0034] Giralt A , Hondares E , Villena JA , Ribas F , Díaz‐Delfín J , Giralt M , Iglesias R , Villarroya F (2011) Peroxisome proliferator‐activated receptor‐γ coactivator‐1α controls transcription of the Sirt3 gene, an essential component of the thermogenic brown adipocyte phenotype. J. Biol. Chem. 286, 16958–16966. 21454513 10.1074/jbc.M110.202390 PMC3089539

[acel12538-bib-0035] Gleyzer N , Vercauteren K , Scarpulla RC (2005) Control of mitochondrial transcription specificity factors (TFB1M and TFB2M) by nuclear respiratory factors (NRF‐1 and NRF‐2) and PGC‐1 family coactivators. Mol. Cell. Biol. 25, 1354–1366. 15684387 10.1128/MCB.25.4.1354-1366.2005 PMC548005

[acel12538-bib-0036] Grollman AP , Moriya M (1993) Mutagenesis by 8‐oxoguanine: an enemy within. Trends Genet. 9, 246–249. 8379000 10.1016/0168-9525(93)90089-z

[acel12538-bib-0037] Guarente L (2008) Mitochondria—a nexus for aging, calorie restriction, and sirtuins? Cell 132, 171–176. 18243090 10.1016/j.cell.2008.01.007 PMC2680180

[acel12538-bib-0038] Hafner AV , Dai J , Gomes AP , Xiao C‐Y , Palmeira CM , Rosenzweig A , Sinclair DA (2010) Regulation of the mPTP by SIRT3‐mediated deacetylation of CypD at lysine 166 suppresses age‐related cardiac hypertrophy. Aging (Albany NY) 2, 914–923. 21212461 10.18632/aging.100252 PMC3034180

[acel12538-bib-0039] Haigis MC , Deng C‐X , Finley LW , Kim H‐S , Gius D (2012) SIRT3 is a mitochondrial tumor suppressor: a scientific tale that connects aberrant cellular ROS, the Warburg effect, and carcinogenesis. Cancer Res. 72, 2468–2472. 22589271 10.1158/0008-5472.CAN-11-3633 PMC3354726

[acel12538-bib-0040] Halaschek‐Wiener J , Amirabbasi‐Beik M , Monfared N , Pieczyk M , Sailer C , Kollar A , Thomas R , Agalaridis G , Yamada S , Oliveira L (2009) Genetic variation in healthy oldest‐old. PLoS One 4, e6641. 19680556 10.1371/journal.pone.0006641 PMC2722017

[acel12538-bib-0041] Hallows WC , Lee S , Denu JM (2006) Sirtuins deacetylate and activate mammalian acetyl‐CoA synthetases. Proc. Natl. Acad. Sci. USA 103, 10230–10235. 16790548 10.1073/pnas.0604392103 PMC1480596

[acel12538-bib-0042] Harper PS (1996) New genes for old diseases: the molecular basis of myotonic dystrophy and Huntington's disease: The Lumbeian Lecture 1995. J. R. Coll. Physicians Lond. 30, 221–231. 8811597 PMC5401426

[acel12538-bib-0043] Hirschey MD , Shimazu T , Goetzman E , Jing E , Schwer B , Lombard DB , Grueter CA , Harris C , Biddinger S , Ilkayeva OR (2010) SIRT3 regulates mitochondrial fatty‐acid oxidation by reversible enzyme deacetylation. Nature 464, 121–125. 20203611 10.1038/nature08778 PMC2841477

[acel12538-bib-0044] Hirschey MD , Shimazu T , Jing E , Grueter CA , Collins AM , Aouizerat B , Stančáková A , Goetzman E , Lam MM , Schwer B (2011) SIRT3 deficiency and mitochondrial protein hyperacetylation accelerate the development of the metabolic syndrome. Mol. Cell 44, 177–190. 21856199 10.1016/j.molcel.2011.07.019 PMC3563434

[acel12538-bib-0045] Hokari F , Kawasaki E , Sakai A , Koshinaka K , Sakuma K , Kawanaka K (2010) Muscle contractile activity regulates Sirt3 protein expression in rat skeletal muscles. J. Appl. Physiol. 109, 332–340. 20413424 10.1152/japplphysiol.00335.2009

[acel12538-bib-0046] Huang K‐H , Hsu C‐C , Fang W‐L , Chi C‐W , Sung M‐T , Kao H‐L , Li AF‐Y , Yin P‐H , Yang M‐H , Lee H‐C (2014) SIRT3 expression as a biomarker for better prognosis in gastric cancer. World J. Surg. 38, 910–917. 24322174 10.1007/s00268-013-2359-0

[acel12538-bib-0047] Iwahara T , Bonasio R , Narendra V , Reinberg D (2012) SIRT3 functions in the nucleus in the control of stress‐related gene expression. Mol. Cell. Biol. 32, 5022–5034. 23045395 10.1128/MCB.00822-12 PMC3510539

[acel12538-bib-0048] Jacobs KM , Pennington JD , Bisht KS , Aykin‐Burns N , Kim H‐S , Mishra M , Sun L , Nguyen P , Ahn B‐H , Leclerc J (2008) SIRT3 interacts with the daf‐16 homolog FOXO3a in the mitochondria, as well as increases FOXO3a dependent gene expression. Int. J. Biol. Sci. 4, 291. 18781224 10.7150/ijbs.4.291 PMC2532794

[acel12538-bib-0049] Jing E , Emanuelli B , Hirschey MD , Boucher J , Lee KY , Lombard D , Verdin EM , Kahn CR (2011) Sirtuin‐3 (Sirt3) regulates skeletal muscle metabolism and insulin signaling via altered mitochondrial oxidation and reactive oxygen species production. Proc. Natl. Acad. Sci. USA 108, 14608–14613. 21873205 10.1073/pnas.1111308108 PMC3167496

[acel12538-bib-0050] John JCS , Facucho‐Oliveira J , Jiang Y , Kelly R , Salah R (2010) Mitochondrial DNA transmission, replication and inheritance: a journey from the gamete through the embryo and into offspring and embryonic stem cells. Hum. Reprod. Update 16, 488–509. 20231166 10.1093/humupd/dmq002

[acel12538-bib-0201] Kawamura Y , Uchijima Y , Horike N , Tonami K , Nishiyama K , Amano T , Asano T , Kurihara Y , Kurihara H (2010) Sirt3 protects in vitro‐fertilized mouse preimplantation embryos against oxidative stress‐induced p53‐mediated developmental arrest. J. Clin. Invest. 120, 2817–2828. 20644252 10.1172/JCI42020 PMC2912189

[acel12538-bib-0051] Kendrick AA , Choudhury M , Rahman SM , McCurdy CE , Friederich M , Van Hove JL , Watson PA , Birdsey N , Bao J , Gius D (2011) Fatty liver is associated with reduced SIRT3 activity and mitochondrial protein hyperacetylation. Biochemical J. 433, 505–514. 10.1042/BJ20100791 PMC3398511 21044047

[acel12538-bib-0052] Kim SC , Sprung R , Chen Y , Xu Y , Ball H , Pei J , Cheng T , Kho Y , Xiao H , Xiao L (2006) Substrate and functional diversity of lysine acetylation revealed by a proteomics survey. Mol. Cell 23, 607–618. 16916647 10.1016/j.molcel.2006.06.026

[acel12538-bib-0053] Kim H‐S , Patel K , Muldoon‐Jacobs K , Bisht KS , Aykin‐Burns N , Pennington JD , van der Meer R , Nguyen P , Savage J , Owens KM (2010) SIRT3 is a mitochondria‐localized tumor suppressor required for maintenance of mitochondrial integrity and metabolism during stress. Cancer Cell 17, 41–52. 20129246 10.1016/j.ccr.2009.11.023 PMC3711519

[acel12538-bib-0054] Kim SH , Lu HF , Alano CC (2011) Neuronal Sirt3 protects against excitotoxic injury in mouse cortical neuron culture. PLoS One 6, e14731. 21390294 10.1371/journal.pone.0014731 PMC3046953

[acel12538-bib-0055] Kincaid B , Bossy‐Wetzel E (2013) Forever young: SIRT3 a shield against mitochondrial meltdown, aging, and neurodegeneration. Front. Aging Neurosci. 5, 1–13. 24046746 10.3389/fnagi.2013.00048 PMC3764375

[acel12538-bib-0056] Kong X , Wang R , Xue Y , Liu X , Zhang H , Chen Y , Fang F , Chang Y (2010) Sirtuin 3, a new target of PGC‐1α, plays an important role in the suppression of ROS and mitochondrial biogenesis. PLoS One 5, e11707. 20661474 10.1371/journal.pone.0011707 PMC2908542

[acel12538-bib-0057] Li D , Ueta E , Kimura T , Yamamoto T , Osaki T (2004) Reactive oxygen species (ROS) control the expression of Bcl‐2 family proteins by regulating their phosphorylation and ubiquitination. Cancer Sci. 95, 644–650. 15298726 10.1111/j.1349-7006.2004.tb03323.x PMC11158795

[acel12538-bib-0058] Li S , Banck M , Mujtaba S , Zhou M‐M , Sugrue MM , Walsh MJ (2010) p53‐induced growth arrest is regulated by the mitochondrial SirT3 deacetylase. PLoS One 5, e10486. 20463968 10.1371/journal.pone.0010486 PMC2864751

[acel12538-bib-0059] Li T , Kon N , Jiang L , Tan M , Ludwig T , Zhao Y , Baer R , Gu W (2012) Tumor suppression in the absence of p53‐mediated cell‐cycle arrest, apoptosis, and senescence. Cell 149, 1269–1283. 22682249 10.1016/j.cell.2012.04.026 PMC3688046

[acel12538-bib-0060] Li H , Feng Z , Wu W , Li J , Zhang J , Xia T (2013) SIRT3 regulates cell proliferation and apoptosis related to energy metabolism in non‐small cell lung cancer cells through deacetylation of *NMNAT2* . Int. J. Oncol. 43, 1420–1430. 24042441 10.3892/ijo.2013.2103 PMC3823398

[acel12538-bib-0061] Lin MT , Beal MF (2006) Mitochondrial dysfunction and oxidative stress in neurodegenerative diseases. Nature 443, 787–795. 17051205 10.1038/nature05292

[acel12538-bib-0062] Lin J , Handschin C , Spiegelman BM (2005) Metabolic control through the PGC‐1 family of transcription coactivators. Cell Metab. 1, 361–370. 16054085 10.1016/j.cmet.2005.05.004

[acel12538-bib-0063] Lombard DB , Alt FW , Cheng H‐L , Bunkenborg J , Streeper RS , Mostoslavsky R , Kim J , Yancopoulos G , Valenzuela D , Murphy A (2007) Mammalian Sir2 homolog SIRT3 regulates global mitochondrial lysine acetylation. Mol. Cell. Biol. 27, 8807–8814. 17923681 10.1128/MCB.01636-07 PMC2169418

[acel12538-bib-0064] Merksamer PI , Liu Y , He W , Hirschey MD , Chen D , Verdin E (2013) The sirtuins, oxidative stress and aging: an emerging link. Aging (Albany NY) 5, 144. 23474711 10.18632/aging.100544 PMC3629286

[acel12538-bib-0065] Miao L , St Clair DK (2009) Regulation of superoxide dismutase genes: implications in disease. Free Radic. Biol. Med. 47, 344–356. 19477268 10.1016/j.freeradbiomed.2009.05.018 PMC2731574

[acel12538-bib-0066] Michishita E , Park JY , Burneskis JM , Barrett JC , Horikawa I (2005) Evolutionarily conserved and nonconserved cellular localizations and functions of human SIRT proteins. Mol. Biol. Cell 16, 4623–4635. 16079181 10.1091/mbc.E05-01-0033 PMC1237069

[acel12538-bib-0067] Miller C , Saada A , Shaul N , Shabtai N , Ben‐Shalom E , Shaag A , Hershkovitz E , Elpeleg O (2004) Defective mitochondrial translation caused by a ribosomal protein (MRPS16) mutation. Ann. Neurol. 56, 734–738. 15505824 10.1002/ana.20282

[acel12538-bib-0068] Morigi M , Perico L , Rota C , Longaretti L , Conti S , Rottoli D , Novelli R , Remuzzi G , Benigni A (2015) Sirtuin 3–dependent mitochondrial dynamic improvements protect against acute kidney injury. J. Clin. Investig. 125, 715. 25607838 10.1172/JCI77632 PMC4319434

[acel12538-bib-0069] Naia L , Rego AC (2015) Sirtuins: double players in Huntington's disease. Biochim. Biophys. Acta 1852, 2183–2194. 26163995 10.1016/j.bbadis.2015.07.003

[acel12538-bib-0070] Nakamura Y , Ogura M , Tanaka D , Inagaki N (2008) Localization of mouse mitochondrial SIRT proteins: shift of SIRT3 to nucleus by co‐expression with SIRT5. Biochem. Biophys. Res. Commun. 366, 174–179. 18054327 10.1016/j.bbrc.2007.11.122

[acel12538-bib-0071] Nguyen LMD , Malamo AG , Larkin‐Kaiser KA , Borsa PA , Adhihetty PJ (2014) Effect of near‐infrared light exposure on mitochondrial signaling in C2C12 muscle cells. Mitochondrion 14, 42–48. 24246911 10.1016/j.mito.2013.11.001

[acel12538-bib-0072] O'Brien TW , O'Brien BJ , Norman RA (2005) Nuclear MRP genes and mitochondrial disease. Gene 354, 147–151. 15908146 10.1016/j.gene.2005.03.026

[acel12538-bib-0073] Palacios OM , Carmona JJ , Michan S , Chen KY , Manabe Y , Ward Iii JL , Goodyear LJ , Tong Q (2009) Diet and exercise signals regulate SIRT3 and activate AMPK and PGC‐1α in skeletal muscle. Aging (Albany NY) 1, 771. 20157566 10.18632/aging.100075 PMC2815736

[acel12538-bib-0074] Park S‐H , Ozden O , Jiang H , Cha YI , Pennington JD , Aykin‐Burns N , Spitz DR , Gius D , Kim H‐S (2011) Sirt3, mitochondrial ROS, ageing, and carcinogenesis. Int. J. Mol. Sci. 12, 6226–6239. 22016654 10.3390/ijms12096226 PMC3189778

[acel12538-bib-0075] Pasinetti GM , Bilski AE , Zhao W (2013) Sirtuins as therapeutic targets of ALS. Cell Res. 23, 1073–1074. 23856645 10.1038/cr.2013.94 PMC3760621

[acel12538-bib-0076] Paulin R , Dromparis P , Sutendra G , Gurtu V , Zervopoulos S , Bowers L , Haromy A , Webster L , Provencher S , Bonnet S , Michelakis ED (2014) Sirtuin 3 deficiency is associated with inhibited mitochondrial function and pulmonary arterial hypertension in rodents and humans. Cell Metab. 20, 827–839. 25284742 10.1016/j.cmet.2014.08.011

[acel12538-bib-0077] Pillai VB , Sundaresan NR , Jeevanandam V , Gupta MP (2010) Mitochondrial SIRT3 and heart disease. Cardiovasc. Res. 88, 250–256. 20685942 10.1093/cvr/cvq250 PMC2952535

[acel12538-bib-0078] Pillai VB , Samant S , Sundaresan NR , Raghuraman H , Kim G , Bonner MY , Arbiser JL , Walker DI , Jones DP , Gius D (2015) Honokiol blocks and reverses cardiac hypertrophy in mice by activating mitochondrial Sirt3. Nat. Commun. 6, 1–16. 10.1038/ncomms7656 PMC4441304 25871545

[acel12538-bib-0079] Qiu X , Brown K , Hirschey MD , Verdin E , Chen D (2010) Calorie restriction reduces oxidative stress by SIRT3‐mediated SOD2 activation. Cell Metab. 12, 662–667. 21109198 10.1016/j.cmet.2010.11.015

[acel12538-bib-0080] Reungwetwattana T , Weroha SJ , Molina JR (2012) Oncogenic pathways, molecularly targeted therapies, and highlighted clinical trials in non–small‐cell lung cancer (NSCLC). Clin. Lung Cancer 13, 252–266. 22154278 10.1016/j.cllc.2011.09.004

[acel12538-bib-0081] Richter C , Park J‐W , Ames BN (1988) Normal oxidative damage to mitochondrial and nuclear DNA is extensive. Proc. Natl Acad. Sci. USA 85, 6465–6467. 3413108 10.1073/pnas.85.17.6465 PMC281993

[acel12538-bib-0082] Rose G , Dato S , Altomare K , Bellizzi D , Garasto S , Greco V , Passarino G , Feraco E , Mari V , Barbi C (2003) Variability of the SIRT3 gene, human silent information regulator Sir2 homologue, and survivorship in the elderly. Exp. Gerontol. 38, 1065–1070. 14580859 10.1016/s0531-5565(03)00209-2

[acel12538-bib-0083] Rutberg S , Saez E , Glick A , Dlugosz A , Spiegelman B , Yuspa S (1996) Differentiation of mouse keratinocytes is accompanied by PKC‐dependent changes in AP‐1 proteins. Oncogene 13, 167–176. 8700543

[acel12538-bib-0084] Satterstrom FK , Swindell WR , Laurent G , Vyas S , Bulyk ML , Haigis MC (2015) Nuclear respiratory factor 2 induces SIRT3 expression. Aging Cell 14, 818–825. 26109058 10.1111/acel.12360 PMC4568969

[acel12538-bib-0085] Scher MB , Vaquero A , Reinberg D (2007) SirT3 is a nuclear NAD+‐dependent histone deacetylase that translocates to the mitochondria upon cellular stress. Genes Dev. 21, 920–928. 17437997 10.1101/gad.1527307 PMC1847710

[acel12538-bib-0086] Schlicker C , Gertz M , Papatheodorou P , Kachholz B , Becker CF , Steegborn C (2008) Substrates and regulation mechanisms for the human mitochondrial sirtuins Sirt3 and Sirt5. J. Mol. Biol. 382, 790–801. 18680753 10.1016/j.jmb.2008.07.048

[acel12538-bib-0087] Schon EA (2000) Mitochondrial genetics and disease. Trends Biochem. Sci. 25, 555–560. 11084368 10.1016/s0968-0004(00)01688-1

[acel12538-bib-0088] Schreiber V , Dantzer F , Ame J‐C , De Murcia G (2006) Poly (ADP‐ribose): novel functions for an old molecule. Nat. Rev. Mol. Cell Biol. 7, 517–528. 16829982 10.1038/nrm1963

[acel12538-bib-0089] Schumacker PT (2010) A tumor suppressor SIRTainty. Cancer Cell 17, 5–6. 20129243 10.1016/j.ccr.2009.12.032

[acel12538-bib-0090] Schumacker PT (2011) SIRT3 controls cancer metabolic reprogramming by regulating ROS and HIF. Cancer Cell 19, 299–300. 21397853 10.1016/j.ccr.2011.03.001 PMC3087169

[acel12538-bib-0091] Schwer B , North BJ , Frye RA , Ott M , Verdin E (2002) The human silent information regulator (Sir) 2 homologue hSIRT3 is a mitochondrial nicotinamide adenine dinucleotide‐dependent deacetylase. J. Cell Biol. 158, 647–657. 12186850 10.1083/jcb.200205057 PMC2174009

[acel12538-bib-0203] Schwer B , Bunkenborg J , Verdin RO , Andersen JS , Verdin E (2006) Reversible lysine acetylation controls the activity of the mitochondrial enzyme acetyl‐CoA synthetase 2. Proc. Natl Acad. Sci. USA 103, 10224–10229. 16788062 10.1073/pnas.0603968103 PMC1502439

[acel12538-bib-0092] Shi T , Wang F , Stieren E , Tong Q (2005) SIRT3, a mitochondrial sirtuin deacetylase, regulates mitochondrial function and thermogenesis in brown adipocytes. J. Biol. Chem. 280, 13560–13567. 15653680 10.1074/jbc.M414670200

[acel12538-bib-0093] Smith BC , Hallows WC , Denu JM (2008) Mechanisms and molecular probes of sirtuins. Chem. Biol. 15, 1002–1013. 18940661 10.1016/j.chembiol.2008.09.009 PMC2626554

[acel12538-bib-0094] Someya S , Yu W , Hallows WC , Xu J , Vann JM , Leeuwenburgh C , Tanokura M , Denu JM , Prolla TA (2010) Sirt3 mediates reduction of oxidative damage and prevention of age‐related hearing loss under caloric restriction. Cell 143, 802–812. 21094524 10.1016/j.cell.2010.10.002 PMC3018849

[acel12538-bib-0095] Song W , Song Y , Kincaid B , Bossy B , Bossy‐Wetzel E (2013) Mutant SOD1 G93A triggers mitochondrial fragmentation in spinal cord motor neurons: neuroprotection by SIRT3 and PGC‐1α. Neurobiol. Dis. 51, 72–81. 22819776 10.1016/j.nbd.2012.07.004 PMC3992938

[acel12538-bib-0096] Sugrue MM , Shin DY , Lee SW , Aaronson SA (1997) Wild‐type p53 triggers a rapid senescence program in human tumor cells lacking functional p53. Proc. Natl Acad. Sci. USA 94, 9648–9653. 9275177 10.1073/pnas.94.18.9648 PMC23243

[acel12538-bib-0097] Sundaresan NR , Samant SA , Pillai VB , Rajamohan SB , Gupta MP (2008) SIRT3 is a stress‐responsive deacetylase in cardiomyocytes that protects cells from stress‐mediated cell death by deacetylation of Ku70. Mol. Cell. Biol. 28, 6384–6401. 18710944 10.1128/MCB.00426-08 PMC2577434

[acel12538-bib-0098] Sundaresan NR , Gupta M , Kim G , Rajamohan SB , Isbatan A , Gupta MP (2009) Sirt3 blocks the cardiac hypertrophic response by augmenting Foxo3a‐dependent antioxidant defense mechanisms in mice. J. Clin. Investig. 119, 2758–2771. 19652361 10.1172/JCI39162 PMC2735933

[acel12538-bib-0099] Sundaresan NR , Bindu S , Pillai VB , Samant S , Pan Y , Huang J‐Y , Gupta M , Nagalingam RS , Wolfgeher D , Verdin E (2016) SIRT3 blocks aging‐associated tissue fibrosis in mice by deacetylating and activating glycogen synthase kinase 3β. Mol. Cell. Biol. 36, 678–692. 10.1128/MCB.00586-15 PMC4760222 26667039

[acel12538-bib-0100] Talos F , Petrenko O , Mena P , Moll UM (2005) Mitochondrially targeted p53 has tumor suppressor activities in vivo. Cancer Res. 65, 9971–9981. 16267022 10.1158/0008-5472.CAN-05-1084

[acel12538-bib-0101] Tanno M , Kuno A , Horio Y , Miura T (2012) Emerging beneficial roles of sirtuins in heart failure. Basic Res. Cardiol. 107, 1–14. 10.1007/s00395-012-0273-5 PMC3390697 22622703

[acel12538-bib-0102] Tao L , Lambert J (2014) The role of sirtuin 3 in the differential pro‐oxidant effects of (‐)‐epigallocatechin‐3‐gallate in oral cells (261.4). FASEB J. 28(261), 264.

[acel12538-bib-0103] Tao R , Coleman MC , Pennington JD , Ozden O , Park S‐H , Jiang H , Kim H‐S , Flynn CR , Hill S , Hayes McDonald W (2010) Sirt3‐mediated deacetylation of evolutionarily conserved lysine 122 regulates MnSOD activity in response to stress. Mol. Cell 40, 893–904. 21172655 10.1016/j.molcel.2010.12.013 PMC3266626

[acel12538-bib-0104] Taylor RW , Turnbull DM (2005) Mitochondrial DNA mutations in human disease. Nat. Rev. Genet. 6, 389–402. 15861210 10.1038/nrg1606 PMC1762815

[acel12538-bib-0105] Tseng AH , Shieh S‐S , Wang DL (2013) SIRT3 deacetylates FOXO3 to protect mitochondria against oxidative damage. Free Radic. Biol. Med. 63, 222–234. 23665396 10.1016/j.freeradbiomed.2013.05.002

[acel12538-bib-0106] Vallejo DM , Caparros E , Dominguez M (2011) Targeting Notch signalling by the conserved miR‐8/200 microRNA family in development and cancer cells. EMBO J. 30, 756–769. 21224847 10.1038/emboj.2010.358 PMC3041954

[acel12538-bib-0107] Virág L , Szabó C (2002) The therapeutic potential of poly (ADP‐ribose) polymerase inhibitors. Pharmacol. Rev. 54, 375–429. 12223530 10.1124/pr.54.3.375

[acel12538-bib-0108] Wang F , Nguyen M , Qin F , Tong Q (2007) SIRT2 deacetylates FOXO3a in response to oxidative stress and caloric restriction. Aging Cell 6, 505–514. 17521387 10.1111/j.1474-9726.2007.00304.x

[acel12538-bib-0109] Wang J‐X , Yi Y , Li Y‐W , Cai X‐Y , He H‐W , Ni X‐C , Zhou J , Cheng Y‐F , Jin J‐J , Fan J (2014a) Down‐regulation of sirtuin 3 is associated with poor prognosis in hepatocellular carcinoma after resection. BMC Cancer 14, 297. 24774224 10.1186/1471-2407-14-297 PMC4021365

[acel12538-bib-0110] Wang X , Wang W , Li L , Perry G , H‐g Lee , Zhu X (2014b) Oxidative stress and mitochondrial dysfunction in Alzheimer's disease. Biochim. Biophys. Acta 1842, 1240–1247. 24189435 10.1016/j.bbadis.2013.10.015 PMC4007397

[acel12538-bib-0111] Wang L , Wang W‐Y , Cao L‐P (2015) SIRT3 inhibits cell proliferation in human gastric cancer through down‐regulation of Notch‐1. Int. J. Clin. Exp. Med. 8, 5263. 26131100 PMC4483974

[acel12538-bib-0112] Weir HJ , Murray TK , Kehoe PG , Love S , Verdin EM , O'Neill MJ , Lane JD , Balthasar N (2012) CNS SIRT3 expression is altered by reactive oxygen species and in Alzheimer's disease. PLoS One 7, e48225. 23139766 10.1371/journal.pone.0048225 PMC3491018

[acel12538-bib-0113] Winnik S , Gaul DS , Siciliani G , Lohmann C , Pasterk L , Calatayud N , Weber J , Eriksson U , Auwerx J , van Tits LJ (2016) Mild endothelial dysfunction in Sirt3 knockout mice fed a high‐cholesterol diet: protective role of a novel C/EBP‐β‐dependent feedback regulation of SOD2. Basic Res. Cardiol. 111, 1–15. 27071400 10.1007/s00395-016-0552-7 PMC4829622

[acel12538-bib-0114] Yan T , Feng Y , Zheng J , Ge X , Zhang Y , Wu D , Zhao J , Zhai Q (2010) Nmnat2 delays axon degeneration in superior cervical ganglia dependent on its NAD synthesis activity. Neurochem. Int. 56, 101–106. 19778564 10.1016/j.neuint.2009.09.007

[acel12538-bib-0115] Yan S‐M , Han X , Han P‐J , Chen H‐M , Huang L‐Y , Li Y (2014) SIRT3 is a novel prognostic biomarker for esophageal squamous cell carcinoma. Med. Oncol. 31, 1–8. 10.1007/s12032-014-0103-8 25005846

[acel12538-bib-0204] Yang H , Yang T , Baur JA , Perez E , Matsui T , Carmona JJ , Lamming DW , Souza‐Pinto NC , Bohr VA , Rosenzweig A , de Cabo R , Sauve AA , Sinclair DA (2007) Nutrient‐sensitive mitochondrial NAD+ levels dictate cell survival. Cell 130, 1095–1107. 17889652 10.1016/j.cell.2007.07.035 PMC3366687

[acel12538-bib-0116] Yang Y , Cimen H , Han M‐J , Shi T , Deng J‐H , Koc H , Palacios OM , Montier L , Bai Y , Tong Q (2010) NAD+‐dependent deacetylase SIRT3 regulates mitochondrial protein synthesis by deacetylation of the ribosomal protein MRPL10. J. Biol. Chem. 285, 7417–7429. 20042612 10.1074/jbc.M109.053421 PMC2844190

[acel12538-bib-0117] Yang B , Fu X , Shao L , Ding Y , Zeng D (2014) Aberrant expression of SIRT3 is conversely correlated with the progression and prognosis of human gastric cancer. Biochem. Biophys. Res. Commun. 443, 156–160. 24287180 10.1016/j.bbrc.2013.11.068

[acel12538-bib-0118] Yin J , Han PC , Caselli R , Beach T , Serrano G , Reiman E , Shi J (2015) Sirtuin 3 is down‐regulated in Apolipoprotein E4 carriers with Alzheimer's disease (P5. 011). Neurology 84, P5.011.

[acel12538-bib-0119] Yoo YA , Kim MJ , Park JK , Chung YM , Lee JH , Chi S‐G , Kim JS , Do Yoo Y (2005) Mitochondrial ribosomal protein L41 suppresses cell growth in association with p53 and p27Kip1. Mol. Cell. Biol. 25, 6603–6616. 16024796 10.1128/MCB.25.15.6603-6616.2005 PMC1190350

[acel12538-bib-0120] Zhang Y‐Y , Zhou L‐M (2012) Sirt3 inhibits hepatocellular carcinoma cell growth through reducing Mdm2‐mediated p53 degradation. Biochem. Biophys. Res. Commun. 423, 26–31. 22609775 10.1016/j.bbrc.2012.05.053

[acel12538-bib-0121] Zhang CZ , Liu L , Cai M , Pan Y , Fu J , Cao Y , Yun J (2012) Low SIRT3 expression correlates with poor differentiation and unfavorable prognosis in primary hepatocellular carcinoma. PLoS One 7, e51703. 23272146 10.1371/journal.pone.0051703 PMC3522714

[acel12538-bib-0122] Zhang B , Qin L , Zhou C‐J , Liu Y‐L , Qian H‐X , He S‐B (2013a) SIRT3 expression in hepatocellular carcinoma and its impact on proliferation and invasion of hepatoma cells. Asian Pac. J. Trop. Med. 6, 649–652. 23790338 10.1016/S1995-7645(13)60112-1

[acel12538-bib-0123] Zhang L , Ren X , Cheng Y , Huber‐Keener K , Liu X , Zhang Y , Yuan Y‐S , Yang JW , Liu C‐G , Yang J‐M (2013b) Identification of Sirtuin 3, a mitochondrial protein deacetylase, as a new contributor to tamoxifen resistance in breast cancer cells. Biochem. Pharmacol. 86, 726–733. 23856293 10.1016/j.bcp.2013.06.032

[acel12538-bib-0124] Zhao Y , Yang H , Wang X , Zhang R , Wang C , Guo Z (2013) Sirtuin‐3 (SIRT3) expression is associated with overall survival in esophageal cancer. Ann. Diagn. Pathol. 17, 483–485. 23871415 10.1016/j.anndiagpath.2013.06.001

[acel12538-bib-0125] Zhao H‐C , Ding T , Ren Y , Li T‐J , Li R , Fan Y , Yan J , Zhao Y , Li M , Yu Y (2016) Role of Sirt3 in mitochondrial biogenesis and developmental competence of human in vitro matured oocytes. Hum. Reprod. 31, 607–622. 26787646 10.1093/humrep/dev345

[acel12538-bib-0126] Zou X , Santa‐Maria C , O'Brien J , Gius D , Zhu Y (2016) MnSOD acetylation and dys‐regulation, due to loss of SIRT3 activity, promotes a Luminal B‐like breast carcinogenic permissive phenotype. Antioxid. Redox Signal. 25, 326–336. 26935174 10.1089/ars.2016.6641 PMC4991597

